# Spatio-temporal evolution and influencing factors of cultural heritage embedded cultural tourism efficiency

**DOI:** 10.3389/fpubh.2025.1690535

**Published:** 2025-12-12

**Authors:** Su Lin, Mengtian Zhang, Linjie Feng, Manwen Lu, Xieqihua Liu

**Affiliations:** 1 Faculty of Humanities and Social Sciences, Macao Polytechnic University, Macao, China; 2 School of Finance and Accounting, Fuzhou University of International Studies and Trade, Fuzhou, China; 3 Institute of Ecological Economy, Lanzhou University of Finance and Economics, Lanzhou, Gansu, China; 4 International Business School, Fuzhou University of International Studies and Trade, Fuzhou, China

**Keywords:** cultural tourism efficiency, cultural heritage, super SBM-DEA, Tobit regression, spatio-temporal evolution

## Abstract

Enhancing the efficiency of cultural tourism serves as a critical catalyst for the synergistic development of culture and the economy. However, existing research has primarily focused on the content of the cultural tourism industry and its coupling coordination relationships, while comparatively little attention has been given to incorporating key resources—such as cultural heritage—into analytical frameworks access efficiency and underlying impact mechanisms. This study introduces an innovative perspective that embeds cultural heritage within the evaluation process. To address the issue of intertemporal and regional comparability, the Super SBM-DEA model is employed to measure the efficiency of cultural heritage embedded cultural tourism (CHECTE) across 30 Chinese provinces from 2012 to 2022 and identifies its driving factors through Tobit regression. The findings demonstrate that average efficiency exhibits a “declining-rising-declining” pattern over the observed period. Specifically, efficiency decreased to 0.5829 in 2015, reached a peak of 0.8511 in 2019, and then experienced a subsequently decline. During periods of decline, efficient areas contract toward the central and western regions, whereas during periods of improvements, these areas expand into the southwest and southeast. This spatial dynamic reveals a notable departure from traditional regional economic gradients. The central region attained a higher average efficiency of 0.7499 compared to the eastern region of 0.5746, suggesting that the central region derives greater benefits from cultural heritage resources than its eastern counterpart. Tobit regression results reveal that transportation conditions, informatization level, policy environment, higher education, and technological innovation are the key driving factors, with significant regional differences. The eastern region is primarily driven by technological innovation and consumption demand, the central region by the policy environment and informatization, and the western region by transportation conditions and higher education. This study offers theoretical and practical guidance for resource allocation and region-specific cultural tourism policies.

## 1 Introduction

The indirect impact of culture on the economy is substantial (1, 2), and the concepts, contents, intellectual properties and other elements nurtured by the cultural industry will overflow to other production sectors, with this effect being especially pronounced in cultural tourism. The quality upgrading of tourism not only requires strengthening service capabilities, but also need to deeply explore historical traditions and national cultural resources to turn them into primary attraction (3). In 2018, China amalgamated the Ministry of Culture with the National Tourism Administration, therefore advancing the integration of culture and tourism to a national strategic priority. In 2019, the document titled “Opinions on Further Stimulating the Potential of Cultural Tourism Consumption” was released. During the Central Economic Work Conference in December 2024, “Expanding service consumption and advancing the cultural tourism industry” was reiterated as a primary objective for 2025. In the same year, China’s Development and Reform Commission, along with seven other departments, collaboratively revised and released the “Implementation Plan for the Cultural Protection, Inheritance and Utilization Project,” clearly stating that national cultural heritage resources should be leveraged to promote high-quality development of cultural tourism.

Consequently, cultural heritage tourism has emerged as a prominent subject of discussion. Leveraging cultural heritage to develop cultural tourism plays a vital role in strengthening regional competitiveness (4). It not only promotes cultural activation, making culture more experiential (5), but also the exoticism embedded in local cultural heritage plays a significant role in motivating tourists (6). Furthermore, studies have shown that the environmental and cultural endowments of World Heritage sites contribute significantly to the development of local tourism (7). As reported by Data Bridge Market Research in 2025, the global cultural heritage tourism market is experiencing steady growth, with the market size expected to reach USD 419.11 billion in 2024 and an estimated compound annual growth rate of up to 5% in the forthcoming years.

While past research has revealed the great energy and value of cultural heritage tourism, the current focus tends to be on content research and industry integration (7–10). This has made it difficult to assess the overall operational efficiency of the sector. Therefore, there is a pressing need to shift the focus toward studying the efficiency of cultural heritage tourism that integrates cultural elements. Efficiency reforms in this context can break the path dependence of industry integration, ultimately optimizing resource allocation within the sector. Sainaghi et al. (11), Wu and Lin (10), and Del Barrio-Tellado et al. (12) have noted that performance evaluation or efficiency assessment represents a highly promising area of research within tourism economics. Some scholars have already proposed interdisciplinary analysis frameworks for efficiency evaluation, demonstrating that the technical efficiency of the tourism industry improves with the involvement of the cultural sector (13). However, questions remain as to whether the resource allocation in cultural heritage tourism has reached an efficient state, and how various regional factors influence this efficiency. These issues require further investigation.

Unlike prior research, this study introduces innovation in the following areas: First, it adopts the embedding of cultural heritage as the primary research perspective. When constructing the efficiency measurement index system, it emphasizes the inclusion of indicators related to cultural heritage resources, cultural spaces, art performances, as well as scenic area resources and tourism supply—factors that significantly influence tourist choices—as the core input variables. Second, regarding the methodology for efficiency assessment, this study utilizes the Super-Efficiency Slack-Based Measure Data Envelopment Analysis (Super-SBM-DEA) model within a global reference framework to evaluate the efficiency of cultural heritage embedded cultural tourism (CHECTE). This approach ensures both temporal and regional comparability of the results. Third, the study takes a multi-dimensional approach in analyzing the influencing mechanisms, considering factors such as infrastructure, policy, society, and technology. This comprehensive analysis provides a solid foundation for identifying bottlenecks and addressing regional coordination challenges in cultural heritage embedded tourism.

The paper is organized as follows: section 2 provides a review of related literature; Section 3 outlines the research design and methods, covering the development of the indicator system and the choice of influencing factors; Section 4 displays the empirical findings, analyzing the temporal and spatial evolution and the driving factors of CHECTE; Section 5 summarizes key conclusions, offers policy suggestions, and discusses limitations along with directions for future research.

## 2 Literature review

### 2.1 The cultural tourism and heritage tourism

Previous studies have consistently demonstrated that the profound integration of culture and tourism is both an unavoidable trend and a necessary pathway for the modernization of the cultural tourism industry (14). Song et al. (15) discovered that cultural-tourism clusters positively influence urban tourism economies, with this impact strengthening once cultural-tourism integration surpasses a certain threshold, additionally generating beneficial spatial spillover effects on neighboring cities. Wang et al. (16) researched the influencing factors of cultural tourism service quality and concluded that, in addition to infrastructure improvements, it is also important to preserve local culture.

Since the inception of interest in the impact of cultural values on tourism, certain experts have identified a significant correlation between the two. Richards (17) further noted that the tourism market is increasingly driven by cultural demand, with cultural creativity transforming traditional, tangible cultural heritage tourism into experiences rooted in intangible culture. As the field continues to evolve, the role of culture and creativity in tourism development has been increasingly significant (18). Guerreiro et al. (19) emphasize that cultural tourism allows visitors to engage creatively through learning about local art, and forming meaningful connections with local cultures. Based on a specialized study, Dai et al. (20) found that cooperation with museums, festival organizers, and other institutions—through the integration of traditional culture with innovative experiences and the establishment of personalized and participatory feedback mechanisms—can significantly enhance tourist satisfaction and attract high-value cultural tourists.

Thus, the preservation of traditional culture remains a central concern. In this context, the mutual embedding of cultural heritage and tourism continues to be a widely discussed and strategically important topic. Trinh and Ryan (6) investigated the demand for heritage tourism, their findings suggest that the exotic sculptural art embedded in the local heritage played a pivotal role in motivating tourists. Similarly, Cuccia et al. (7) examined the impact of World Heritage List designations on tourism development in Italy. Their study concluded that the environmental and cultural assets inherent in local heritage sites significantly contribute to the region’s tourism growth. Du et al. (21) investigated the link between intangible cultural heritage and tourism development, finding that the coupling and coordination between them improved from 2013 to 2022.

A growing body of research affirms that public demand for cultural heritage tourism is strong, and this demand serves as a key driver of tourist motivation and destination attractiveness. Pan et al. (22) argued that the integration of the heritage economy, the cultural economy, and the tourism economy leads to the emergence of an interdisciplinary framework known as the heritage economy. Although a clear conceptual model for the heritage and cultural tourism economy is still evolving, cultural heritage tourism is increasingly recognized as a strategy for preserving specific heritage and regional development (23). In this vein, Ma et al. (24) found that rural tourism rooted in both tangible and intangible cultural heritage promotes diversification of household livelihood strategies while enhancing local financial, human, and social capital, thereby aiding in the preservation of traditional culture. Li & Zhong (25) argued that ancient villages renowned for their unique cultural heritage have become important areas with tourism value, and attempted to create a development model that balances culture and tourism based on the concept of smart villages.

### 2.2 The measurement of cultural tourism efficiency

Data Envelopment Analysis (DEA) is a non-parametric method employed to evaluate relative efficiency by measuring how far Decision-Making Units (DMUs) deviate from the production frontier. Nurmatov et al. (26) reviewed the application of DEA models in the tourism and hospitality industry. Their analysis revealed that 52.13% of the studies adopted traditional DEA models, 11.15% utilized bootstrap DEA models, and 8.52% employed slacks-based DEA models, highlighting the methodological evolution within the field.

In terms of the application of different DEA models, Corne (27) used the hierarchical category DEA model to analyze the technical efficiency of the French hospitality industry, concluding that economy hotels exhibit greater efficient than other categories; Cuccia et al. (7) demonstrated the beneficial effect of the cultural and natural assets encompassed in the World Heritage Sites on the tourism efficiency of Italian regions, employing a two-stage DEA model; Niavis and Tsiotas (28) studied the efficiency of tourism destinations along the Mediterranean coast from 2015 to 2017 using traditional DEA; Wu and Lin (10) evaluated the cultural tourism performance of Asian tourist destinations through a dynamic DEA approach; Jeon et al. (29) used DEA to calculate scale efficiency to measure the efficiency of global geoparks and their impact on the surrounding economy, filling a gap in efficiency research on this type of tourist destination.

Some scholars have also focused on Chinese regions as the primary units of analysis. Chaabouni (30) employed a two-stage double-bootstrap DEA approach to analyze tourism efficiency and its determinants across 31 Chinese provinces from 2008 to 2013, found that trade openness, climate variation, and market competition have improved tourism efficiency. Similarly, Song and Li (31) utilized both traditional DEA and bootstrap DEA models to estimate tourism efficiency across 31 provinces from 2011 to 2016. The study found that economic development, urbanization, and openness to international markets exerted significant positive effects on tourism efficiency. Wang et al. (32) conducted a related study on the tourism efficiency of 30 Chinese provinces over the same 2011–2016 period, employing a Super-DEA model. Their findings indicated a slight decline in overall tourism efficiency during the study period, and they recommended strengthening tourism cooperation mechanisms to enhance efficiency. Zhang and Wu (33) employed a three-stage DEA model to assess tourism efficiency in 22 cities along the Grand Canal. Their analysis revealed that areas with well-developed tourism tend to show an “H-H” distribution pattern, whereas less developed regions are characterized by “H-L” or “L-L” patterns.

### 2.3 Research reviews

As previously noted, existing research consistently underscores the critical role of cultural heritage in regional tourism development and its capacity to meet public demand for heritage tourism. Investigations into the synergy within the cultural tourism industry have demonstrated the positive influence of cultural resources on tourism efficiency and adjacent areas. However, the specific efficiency of cultural heritage embedded cultural tourism (CHECTE) has received limited scholarly attention. Addressing efficiency-related issues is vital for the ongoing optimization of resource allocation in industrial integration, thereby necessitating a focused analytical approach in this domain. Previous provincial-level studies in China primarily examined tourism efficiency drivers such as economic development and openness, highlighting coastal advantages. However, they have not revealed the different spatiotemporal evolution patterns of CHECTE, nor the heterogeneity of driving factors. Methodologically, while traditional DEA models and their variants dominate tourism efficiency assessments, few studies adopt long-term globally comparable benchmarks, risking intertemporal comparability limitations. To address this, our research employs a Super-SBM-DEA model with a global reference framework, seeking to enhance the robustness and cross-spatiotemporal comparability of CHECTE.

## 3 Research design and methodology

### 3.1 Study area and data sources

**Figure 1:**
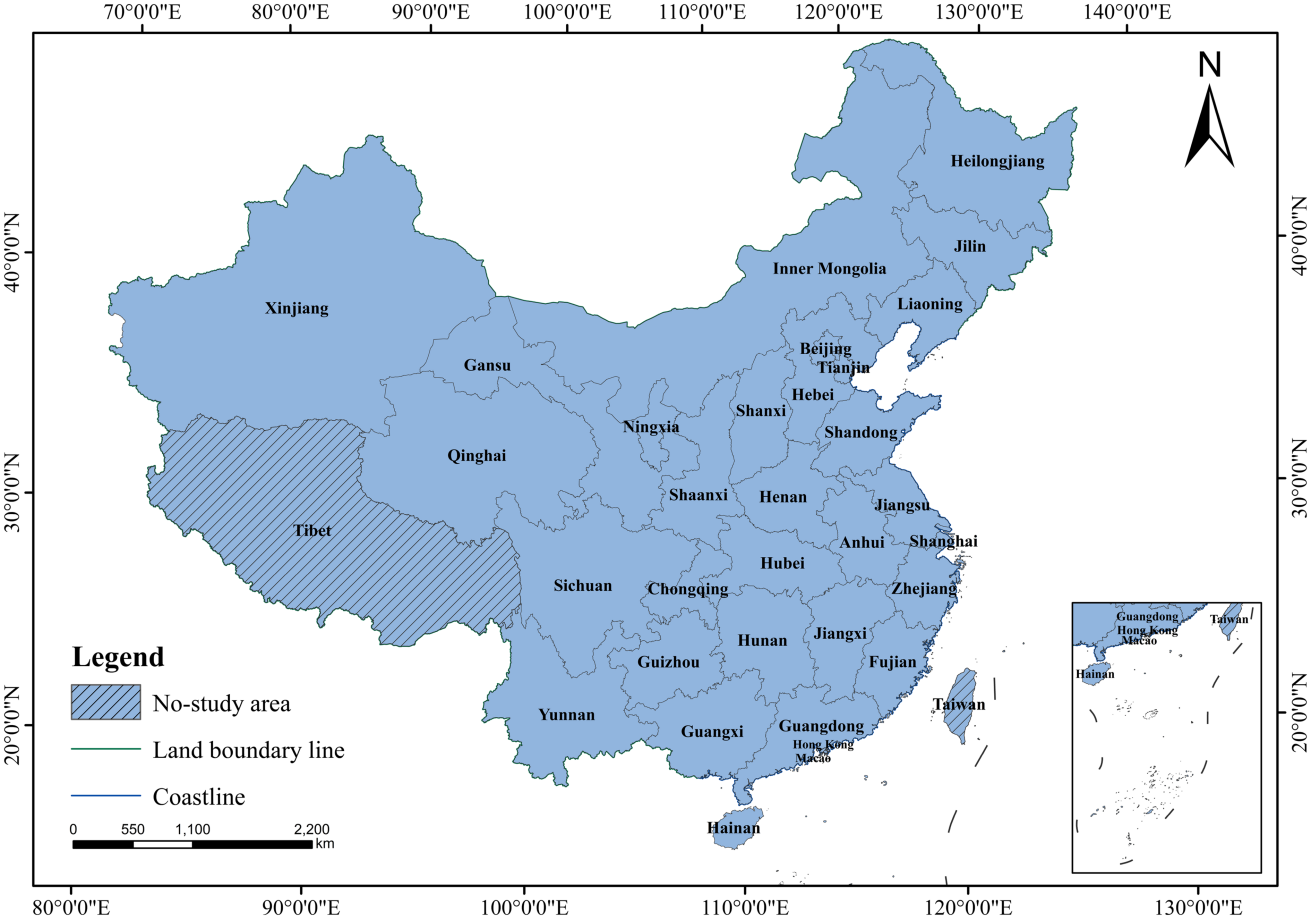
Study area the base map is based on the standard map of the Ministry of Natural Resources [Map approval number: GS (2023)2764], with no modification of boundaries. Data for Tibet, Hong Kong, Macao, and Taiwan are currently unavailable. The same convention applies hereafter.

Based on the data availability, the data of 30 provinces except Tibet, Hong Kong, Macao and Taiwan were selected as samples, and referring to the regional division of the National Bureau of Statistics, the 10 eastern provinces and municipalities and the 3 northeastern provinces were collectively referred to as the eastern region—Beijing, Tianjin, Hebei, Shandong, Jiangsu, Shanghai, Zhejiang, Fujian, Guangdong, Hainan, Heilongjiang, Jilin, Liaoning. While the central region included 6 provinces—Shanxi, Henan, Hubei, Hunan, Anhui, Jiangxi. The western region included 12 provinces, municipalities and districts—Inner Mongolia, Guangxi, Chongqing, Sichuan, Guizhou, Yunnan, Shaanxi, Gansu, Qinghai, Ningxia, Xinjiang. And the study area is shown in Figure 1.

The inaugural edition of the “China Statistical Yearbook on Culture and Related Industries” dates back to 2013 and was subsequently rebranded as the “China Statistical Yearbook on Culture, Cultural Relics and Tourism” following the amalgamation of the culture and tourism sectors. The most recent yearbook is the 2023 edition, this study constructs a panel dataset for 30 provinces covering the period from 2012 to 2022. The data is sourced from the China Tourism Statistical Yearbook, the National Bureau of Statistics, public records from provincial departments of culture and tourism, and the National Economic and Social Development Statistical Bulletin, among others. Variable indicators related to pricing are calibrated to the base period of 2012 constant prices.

### 3.2 Global reference super-SBM-DEA for efficiency assessment

#### 3.2.1 Overview of the model

Production theory conceptualizes economic systems as transformation processes that convert various input factors into corresponding output outcomes (33, 34). Tourism centered on cultural heritage embedded tourism exhibits the characteristics of complex system involving multiple inputs and outputs, thereby aligning well with this theoretical framework. To quantify conversion efficiency, the Data Envelopment Analysis (DEA) model was employed. This model has become a prominent analytical tool in efficiency research due to its objectivity and minimal requirement for prior assumptions (35). DEA has been widely applied to assess tourism efficiency (10, 29) and is particularly prevalent in studies examining provincial-level tourism efficiency in China (30, 31). Traditional DEA models typically rely on radial and angular measurements, which fail to account for input and output slack, potentially leading to biased efficiency evaluations. However, improvements in inputs, such as cultural resources and human capital, and in outputs, such as visitor numbers and tourism revenue, are often disproportionate within the cultural tourism sector. To address this limitation, Tone (36) proposed the non-radial and non-angular Slack-Based Measure of DEA (SBM-DEA) model, thus providing a more accurate representation of efficiency by incorporating slack variables and minimizing bias. Building upon this framework, the Super-SMB-DEA model further enables the comparison and ranking of decision-making units (DMUs) that are deemed efficient. This extended model has been successfully applied and empirically validated in sectors characterized by complex production structures (32, 37, 38). However, most studies utilizing these models have not incorporated the time factor or examined the dynamic evolution of efficiency over the long term, leaving a gap in understanding how efficiency characteristics evolve.

Therefore, this study employs the global reference Super-SBM-DEA model, which constructs the frontier using data from all years, thereby facilitating the comparability of intertemporal efficiencies, mitigating misjudgments caused by technological regressions, and is appropriate for the analysis of long-term panel data. Simultaneously, super-efficiency measurement can pinpoint efficient provinces, allowing for comparative analysis among them, thereby addressing the traditional DEA’s inability to distinguish between efficient units with an efficiency exceeding 1 and the inter-period technical variability issue. The model is shown in Equation 1:


$$\rho^{G*}=\min\frac{1-\frac{1}{m}\sum_{i=1}^{m}\frac{s_{i}^{-}}{x_{\mathrm{ik}}^{t}}}{1+\frac{1}{q}\sum_{r=1}^{q}\frac{s_{r}^{+}}{y_{\mathrm{rk}}^{t}}}$$

s.t.


$$\begin{array}{c} \sum_{t=1}^{T}\;\sum_{j=1,j\neq k}^{n}\;\lambda_{j}^{t}x_{\mathrm{ij}}^{t}+s_{i}^{-}=x_{\mathrm{ik}}^{t},(i=1,,,2,,,\ldots ,,,m)\\ \sum_{t=1}^{T}\;\sum_{j=1,j\neq k}^{n}\;\lambda_{j}^{t}y_{\mathrm{rj}}^{t}-s_{r}^{+}=y_{\mathrm{rk}}^{t},(r=1,,,2,,,\ldots ,,,q) \end{array}$$

(1) $$\lambda_{j}^{t}\geq 0,s_{i}^{-}\geq 0,s_{r}^{+}\geq 0,(i=1\ldots m;r=1,\ldots ,s;t=1,\ldots ,T;j=1,\ldots ,n)$$

The numerator of the objective function reflects the proportion of input improvement, and the denominator reflects the proportion of output improvement. If $\rho^{G*}$ is greater than 1, it indicates that the unit’s efficiency is superior to the global frontier; if it is equal to 1, it indicates that it is at the global efficiency frontier; both indicate that the resource allocation has reached the efficiency state; m, q indicates the number of input and output indicators, respectively; $s_{i}^{-}$ is the amount of redundancy of inputs, $s_{r}^{+}$ is the amount of output slacks. $x_{\mathrm{ik}}^{t},y_{\mathrm{rk}}^{t}$ indicate the i input and r output value of the kth DMU (i.e., the province) of the period t, respectively. T is the number of periods, n is the number of DMUs, and $\lambda_{j}^{t}$ is the weight coefficient, reflecting the reference contribution of the jth decision unit in each period in constructing the frontier. The efficiency results are the overall efficiency based on the assumption of constant returns to scale.

#### 3.2.2 The index system of CHECTE

Accurate efficiency measurement requires incorporating the core input and output variables that impact the development efficiency of cultural tourism into the indicator system. Some scholars have researched tourism and its supply chain, proposing that the effectiveness of tourism promotion is related to the adequacy of resources and supply chains, and that resources such as museums, historical heritages, scenic spots, etc. should be taken into account (39, 40), and that the number of local star-rated hotels and travel agencies can be taken as the capital inputs for the supply and service of tourism services (35). Since culture-seeking tourists can bring more income to the local area than other tourists (41), tourism income serves as an important output measure.

This paper focuses on the performance of the CHECTE. Relevant studies suggest that in constructing indicators for cultural tourism efficiency, it is necessary to consider not only factors such as the number of museums, cultural heritage sites, and the protection efforts of these sites (35, 42), but also the broader scope of cultural tourism. Some scholars emphasize that cultural tourism encompasses not only heritage tourism, such as visits to historical sites, but also ethnic tourism, including cultural expressions like dances, performances, and rituals (8). Intangible cultural heritage, under rational management, can be one of the most valuable tourism assets (43). The integration of cultural creativity with tangible heritage can convert more intangible culture into new forms of tourism (17). This fusion of cultural creativity and tangible heritage drives the diversification of cultural tourism, including performances and artistic activities. Research has shown that an increase in artistic events is positively associated with the growth in cultural tourism (44).

The above studies show that the embedding of cultural heritage and other resources directly affects the diversity of cultural tourism forms, and the input indicators not only need to have factors such as heritage sites, heritage places, and public spaces of museums, but also need to take into account intangible cultural heritage, art performances, and special cultural tourism destinations. Base on that, the input indicators mainly include resources, capital, and labor input, especially focusing on the embedding of cultural heritage resources. Output indicators are mainly reflected by market effects, mainly centered on dimensions such as audience attendance and income, focusing on how to effectively utilize these resources to generate more audiences, tourists and income. The whole indicators are shown in Table 1.

**Table 1:** Indicator system of CHECTE.

Dimension	Secondary indicators	Tertiary indicators (Unit)	Reference sources
Input Indicators	Resource input	Number of national key cultural relics protection units	Wu and Lin. (10) and Xu et al. (49)
		Number of intangible cultural heritage (items)	Xu et al. (49)
		Number of A-class scenic spots	Song and Li. (31) and Xu et al. (49)
		Number of Leisure Agriculture and Rural Tourism Demonstration Counties (Sites)	–
	Capital investment	Number of museums	Xu et al. (49) and Lu et al. (5)
		Number of performing arts groups	Lu et al. (5)
		Number of star-rated hotels	Xu et al. (49), Wang et al. (35) and Chen et al. (45)
		Number of travel agencies	Chen et al. (45), Xu et al. (49) and Wang et al. (35)
	Labor input	Number of employees in the tourism industry (10,000 persons)	Chaabouni (30), Wang et al. (35) and Chen et al. (45)
		Number of employees in the culture, sports and entertainment industry (10,000 persons)	Lu et al. (5)
Output indicators	Market effect	Visits to museums (10,000 visits)	Xu et al. (49)
		Attendance of performing arts groups (10,000 attendances)	Huang et al. (54)
		Total number of tourists (10,000 person-times)	Chaabouni, (30), Song and Li. (31) and Xu et al. (49)
		Gross Tourism Income (100 million RMB)	Wang et al. (35), Chen et al. (45), and Wu and Lin (10)

### 3.3 Tobit regression analysis of influencing factors

#### 3.3.1 Model overview

Since the super-efficiency measurements are between 0 and 1.2371 for truncated data, with a left cut-off point of 0, the Tobit model is introduced to explore the influencing factors of CHECTE. This model is shown in Equation 2:


$$\rho_{\mathrm{it}}^{G*}=\beta_{0}+\sum_{k=1}^{n}\beta_{k}X_{\mathrm{kit}}+\mu_{i}+\lambda_{t}+\varepsilon_{\mathrm{it}}$$

(2) $$\rho_{\mathrm{it}}^{G}=\{\begin{array}{ll} \rho^{G*} & \text{if}\;\rho^{G*}\geq 0\\ 0 & \text{if}\;\rho^{G*}<0 \end{array}$$

Where: i denotes the cross-sectional unit, in this study it is the province, and t denotes the year; $\rho_{\mathrm{it}}^{G*}$ is the observed explanatory variable, i.e., the value of hyper-efficiency of cultural tourism development of the ith province in the tth year; $\rho_{\mathrm{it}}^{G}$ is the potential explanatory variable, and the rules of taking the value are shown in the formula; $\;X_{\mathrm{kit}}$ is the value of the kth explanatory variable in the tth year of the ith unit, and n is the number of explanatory variables; $\beta_{0}$ is the constant term; $\beta_{k}$ is the regression coefficient of the kth explanatory variable, $\mu_{i},\lambda_{t}\;$ is the individual fixed effect and time fixed effect, respectively; $\varepsilon_{\mathrm{it}}$ is the error term.

#### 3.3.2 Selection of influencing factors

Grounded in the evolution of production theory from classical to neoclassical and modern economics, this study adopts an extended production function framework. This framework transcends the conventional factors like capital and labor, which are directly measured in the Super-SBM-DEA model, and conceptualizes the CHECTE as being critically influenced by external environmental and soft factors. These factors encompass physical and digital infrastructure, controller, driving pull, and the intellect and engine of the system, collectively enhancing or impeding efficiency through their impact on resource allocation and output optimization.

**Transportation Conditions (TC).** Transportation has an obvious impact on the choice of destination: On the one hand, convenient and developed transportation is a prerequisite for prosperity, it can eliminate the spatial barriers to the development of cultural tourism, accelerate the aggregation and diffusion effect of industrial elements; on the other hand, excellent transportation conditions will amplify the centripetal effect of surrounding industry elements, which may exacerbate the imbalance among regions (5). As a result, regional transportation may either promote or impede the efficiency of culture tourism, according to related studies that use the carrying capacity of a region’s core transportation network, such as railroads and highways, to assess transportation conditions (45, 46).

**Degree of Informatization (IN).** The degree of informatization has become a significant form of infrastructure investment that influences tourists’ perceptions and choice (35). This impact is more pronounced in the digital era. Some regions may lack environment advantages, however, they can still stimulate the growth of new cultural tourism industries by enhancing digital infrastructure, such as expanding Internet broadband and fiber-optic networks. The development of digital infrastructure is typically assessed using indicators like the total length of fiber-optic cable lines (47). Accordingly, the per capita length of fiber-optic cables is selected to represent the degree of informatization.

**Policy Environment (PE).** Effective development and conservation of cultural resources significantly impact cultural tourism growth (48). Studies reveal that an increase in arts events is associated with a rise in cultural tourism (44), with arts development often requiring government planning, coordination, and promotion. Therefore, the policy environment reflected by government expenditures on cultural tourism, sports, and media, plays a crucial role. Per capita spending in these areas can serve as a representative indicator (5). However, excessive investments may lead to redundancy and inefficiency, making the exact direction of impact an area that requires further investigation.

**Consumption Demand (CD).** The expansion of the economy drives greater consumption of cultural and recreational activities, leading to boosted investment and infrastructure development in local cultural tourism, which in turn improves its efficiency (5). Xu et al. (49) also highlight that economic factors and consumption levels substantially affect the integration of cultural tourism. To measure this, per capita cultural and recreational consumption is commonly used as an indicator of consumption demand.

**Higher Education Level (HE).** Education level is a common determinant of efficiency across various fields, including cultural tourism. Yolal and Negrusa (50) showed that young, highly educated individuals are the primary consumers of cultural tourism products. Furthermore, areas with higher educational attainment generally exhibit more efficient cultural heritage organizations, especially museums, which influence both cultural production and visitor behavior (12). Thus, the average count of higher education enrollees per 100,000 individuals acts as a key proxy for assessing regional education levels.

**Technology Innovation (TI).** Several scholars found that a region’s natural attractions must be complemented by cultural ones that pique tourists’ curiosity. This can be achieved by developing network technologies to intelligently access local specific cultural information (9). Technology innovation is an important driver of cultural tourism productivity. Wang and Yang (51) examined the development of cultural tourism through qualitative comparative analysis, finding that productivity driven by digital technological innovation is essential for achieving high levels of cultural tourism development. Therefore, per capita financial expenditures on science and technology are used as an indicator of technological innovation.

Based on the above, the influencing factors and variables measurement are summarized in Table 2.

**Table 2:** Influential factors and variables selected.

Influencing factors	Variable connotation	Indicator
Transportation conditions (TC)	Infrastructure	Railroad and road transportation capacity (billions of people)
Degree of informatization (IN)	Digital infrastructure	Length of fiber optic cable lines per 100 people (km)
Policy environment (PE)	Controller	Per capita expenditure on culture, tourism, sports, and media (100 yuan)
Consumption demand (CD)	Driving pull	Per capita cultural and recreational consumption (100 yuan)
Higher education level (HE)	Intellect	Average number of students enrolled in higher education per 100,000 population (100)
Technology innovation (TI)	Engine	Per capita financial expenditure on science and technology (100 yuan)

**Figure 2:**
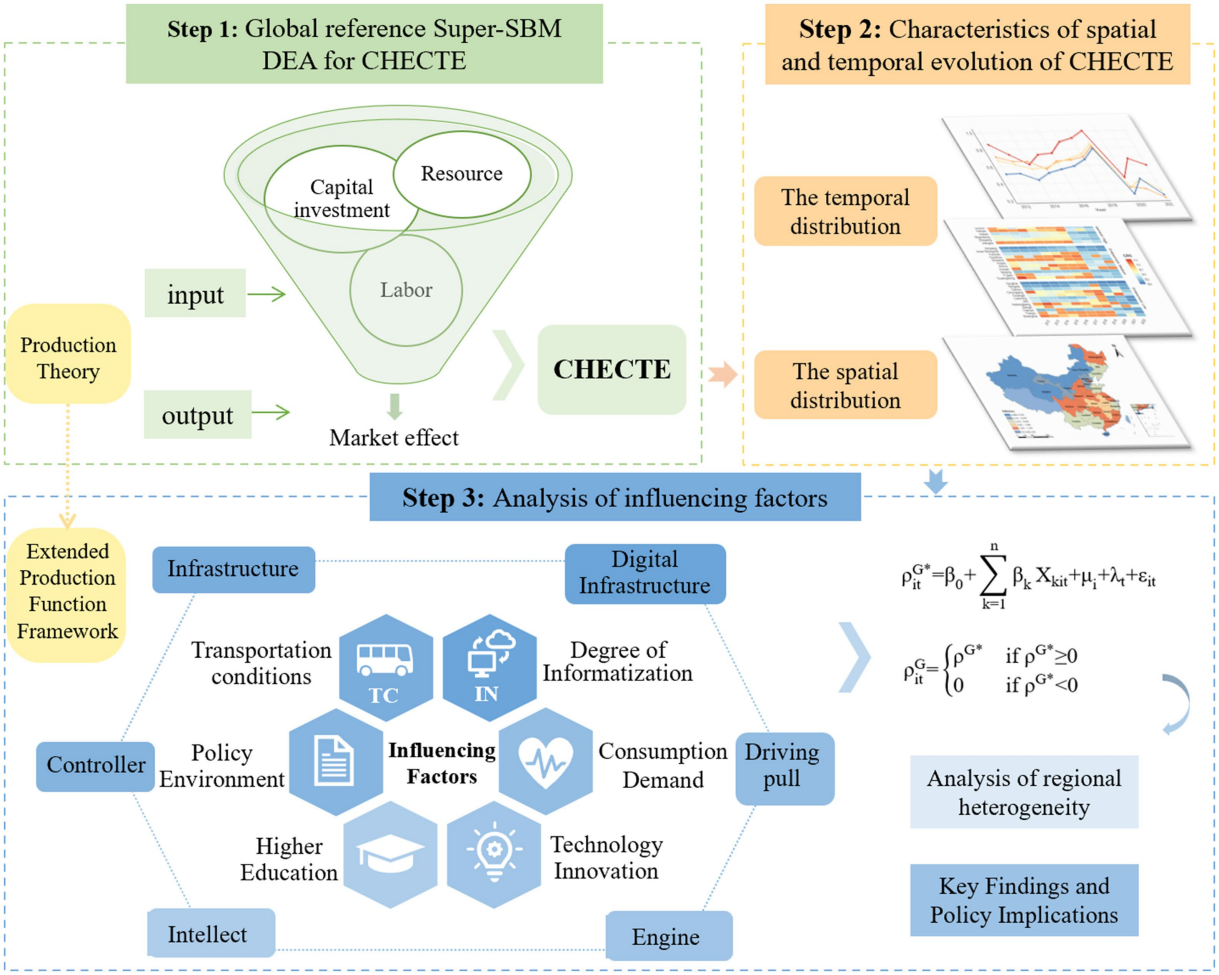
Theoretical Framework and Research Flowchart.

Based on the aforementioned research design, theoretical foundations, and methodological analysis, the theoretical framework and research flowchart for this study were constructed (Figure 2).

## 4 Results and discussion

### 4.1 Characteristics of spatial and temporal evolution of CHECTE

#### 4.1.1 The temporal distribution of CHECTE

**Figure 3:**
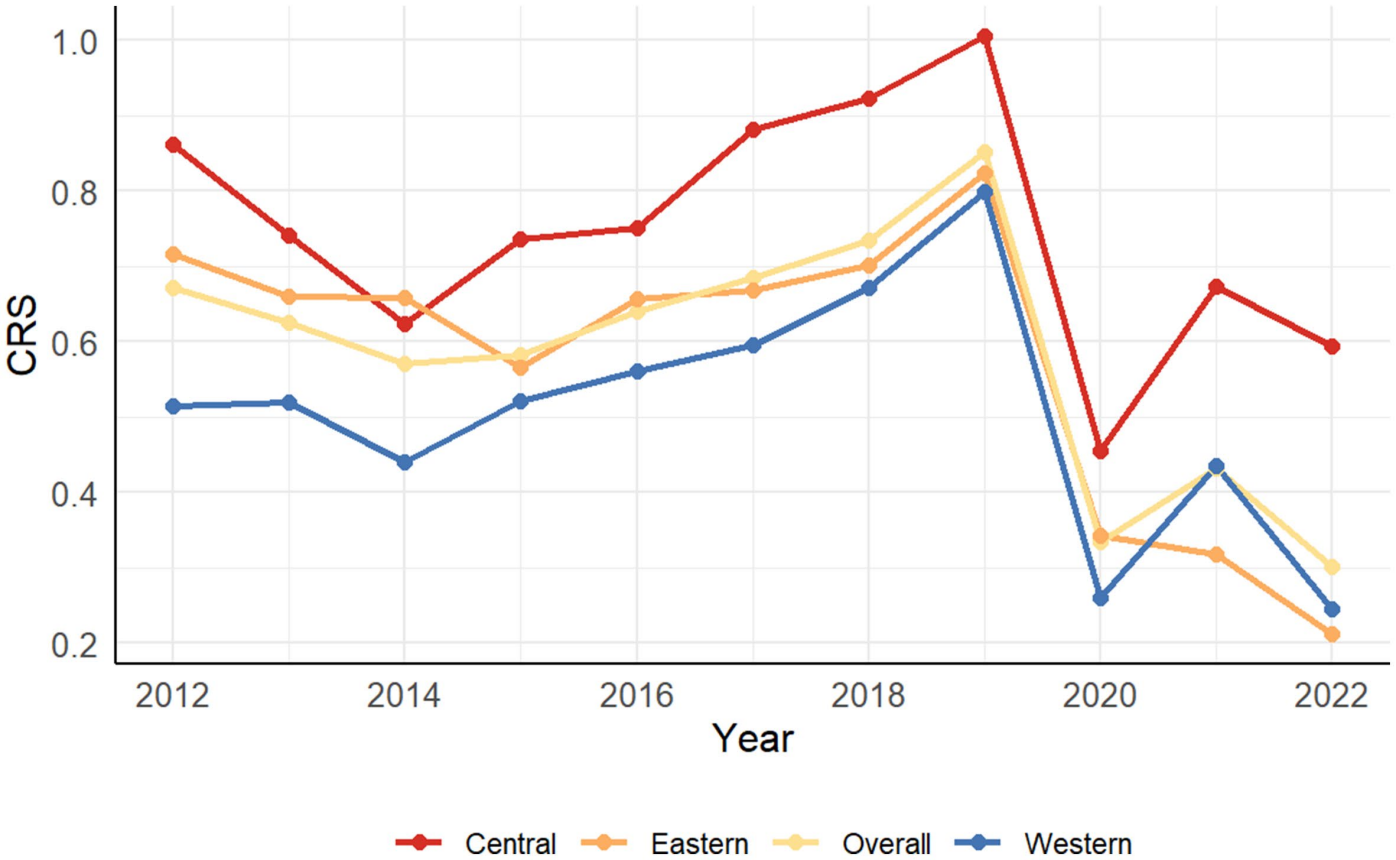
The average trend of CHECTE in 4 regions (2012–2022).

The regional divisions of the 30 provinces are as described in the research area mentioned earlier. The line chart depicting the average CHECTE values across these regions is presented in Figure 3. The trend reveals a “decline–rise–decline” pattern. This trajectory aligns with the findings of Lu et al. (5), Wang et al. (32), and other scholars, indirectly validating the rationality of the selected indicators.

Initiated in 2011, China’s 12th Five-Year Plan formally recognized “cultural tourism integration” as a developmental pathway. However, transformative policies exhibited a time lag in their effectiveness. Entering the 13th Five-Year Plan in 2016, new concepts and business models such as “regional tourism” and “culture-tourism integration” began gaining prominence. In 2017, regional tourism was elevated to a national strategy, tourism development increasingly relied on cultural heritage resources; culture-tourism integration was institutionalized in 2018, and was formally endorsed as a key driver of consumption by the State Council in 2019.

Therefore, as evidenced by the aforementioned policy evolution, cultural and tourism resources prior to the 13th Five-Year Plan remained fragmented across different administrative departments, hindering resource coordination. Consequently, heritage tourism and folk culture frequently failed to translate effectively into tourism appeal. Furthermore, before the implementation of pivotal initiatives like the “Toilet Revolution” in 2015, this phase was characterized by deficiencies in public services and infrastructure. Consequently, overall efficiency declined from 0.6713 to 0.5829 during this stage.

Following the implementation of relevant strategies in 2016, cultural and tourism development efficiency values rose steadily across multiple regions. By 2019, most provinces achieved CHECTE above 1, indicating efficient resource allocation. This illustrates the strong policy-driven nature of the sector, where institutional reform and management innovation facilitated a more fluid and effective distribution of resources. Thus, the period from 2016 to 2019 constituted a phase of stable growth fueled by policy dividends, with the average efficiency reaching a peak of 0.8511 in 2019.

Following the onset of the COVID-19 pandemic in 2020, CHECTE experienced a pronounced decline, with the average efficiency plummeting from 0.8511 to 0.3342. A modest recovery emerged in 2021, yet only four provinces—Hunan, Jiangxi, Guizhou, and Guangxi—achieved efficiency values exceeding 1, while 22 provinces remained below 0.5. The Northwest region exhibited vulnerability, with Qinghai and Ningxia sustaining values below 0.2, underscoring the limited resilience of many provinces against external shocks.

#### 4.1.2 The spatial distribution of CHECTE

**Figure 4:**
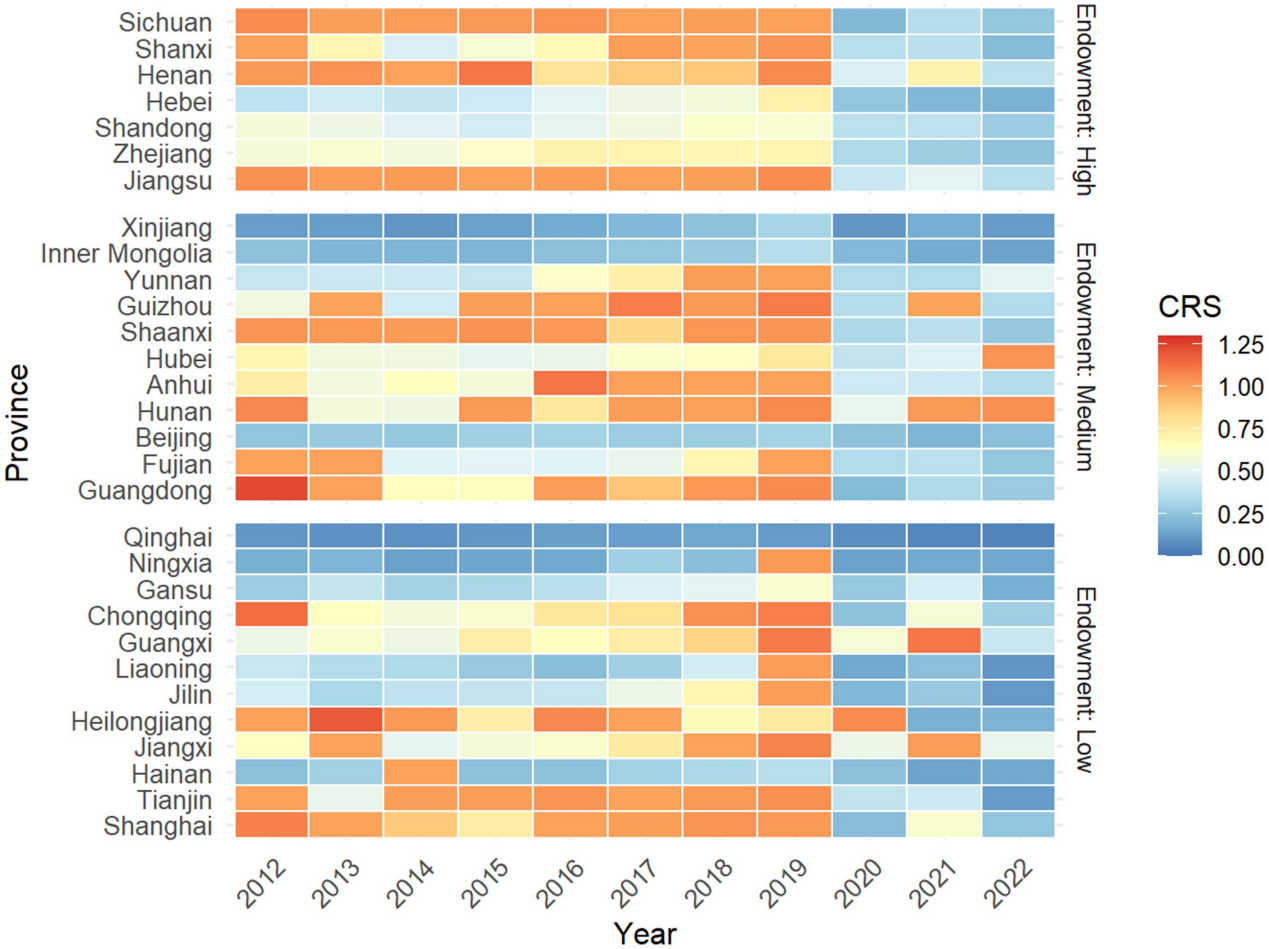
Heat map of CHECTE in each province.

Figure 4 displays the value of CHECTE in each province. The number of national key cultural heritage protection units and national intangible cultural heritage owned by each province is used to reflect the characteristics of its cultural heritage endowment, and each module is from top to bottom for the provinces in the western, central, and eastern regions, respectively. It could be seen that the high and low endowment of cultural heritage resources cannot directly match the efficiency characteristics of cultural tourism development in each province. Among the provinces with high cultural heritage endowment, Sichuan in the west, as well as Shanxi and Henan in the center, are able to achieve perennial efficiency leadership, but some eastern provinces are not close to the high efficiency status. Chongqing, Guangxi, Tianjin, Shanghai and other provinces with low endowment of cultural resources have achieved high efficiency through good operation of the cultural tourism industry, reflecting their outstanding ability to integrate cultural tourism resources and market operation. Against the background of the same low endowment, the efficiency values of western provinces such as Qinghai, Ningxia and Gansu have been low for a long period of time, which shows that in addition to resource disadvantages, such areas may also be limited by factors such as infrastructure.

**Figure 5:**
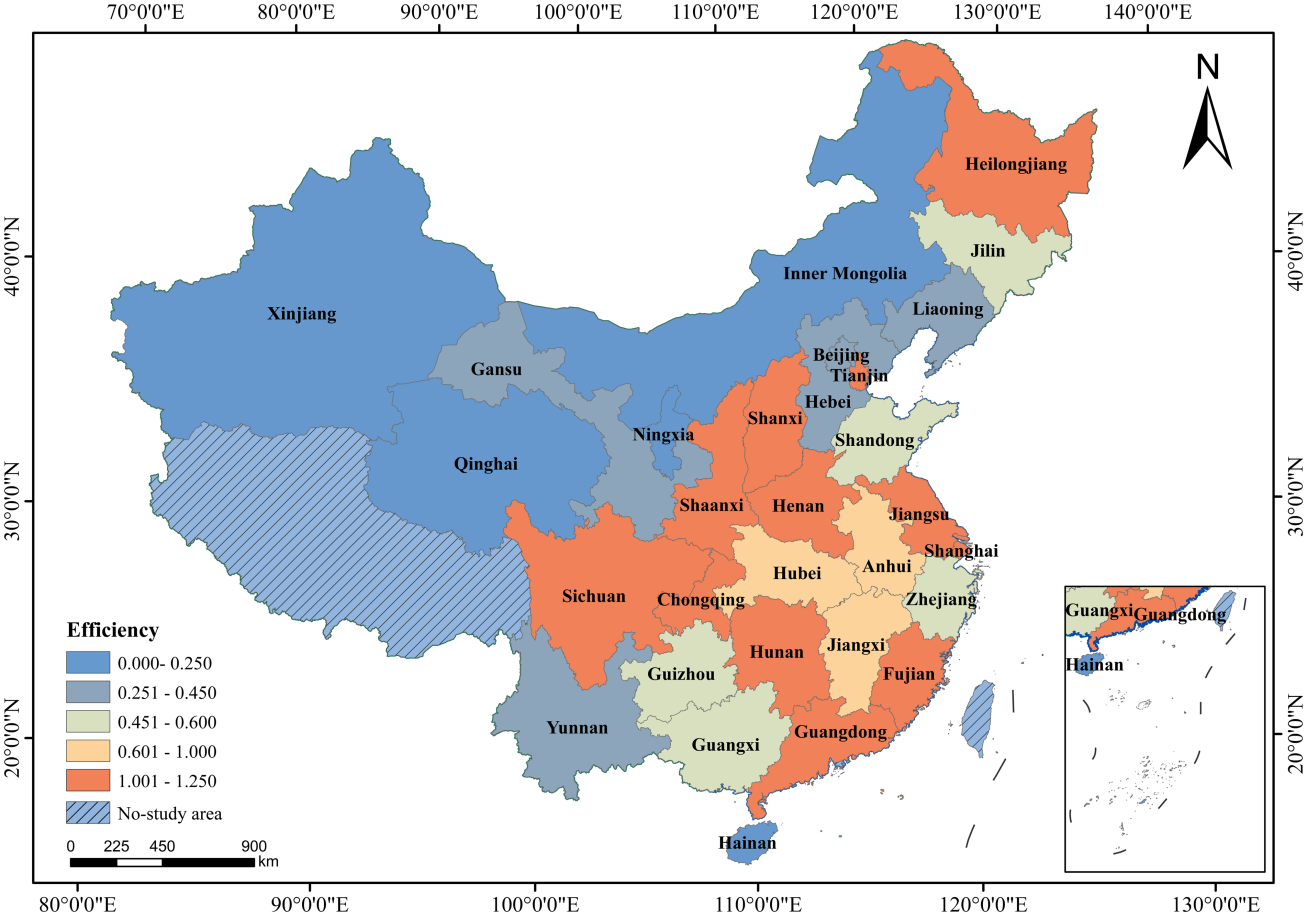
Distribution of the average value of CHECTE by province (2012–2022).

ArcGIS is used to map the distribution of the average efficiency of cultural heritage embedded cultural tourism in each province, and the results are shown in Figure 5. During the study period, the average efficiency characteristics did not align with the geographical distribution of economic development. The central and western provinces benefited more from cultural heritage than the eastern provinces, which aligns with existing research and the latest findings by Chen et al. (45, 52). In terms of average efficiency, the overall mean efficiency value is 0.5843, with the central region attaining a higher level of 0.7499—surpassing the eastern region’s average of 0.5746. CHECTE exhibits new patterns. The average efficiency distribution chart shows that, despite the strong economic foundation and high marketization of the cultural tourism industry in many eastern provinces, their overall efficiency in integrating cultural resources does not significantly outperform that of the central and western regions. In contrast, some provinces in central and western China benefit from better cultural heritage resources or supportive policies, such as the “Yellow River Golden Triangle Region of Shanxi-Shaanxi-Henan,” which enhance efficiency. Western China, rich in ecological and cultural resources, faces transportation challenges that hinder development, particularly in northwest China, where average efficiency is the lowest. In this case, infrastructure could play a key positive role. It is evident that the impact and magnitude of various factors vary across regions, which will be further explored and empirically tested later.

**Figure 6:**
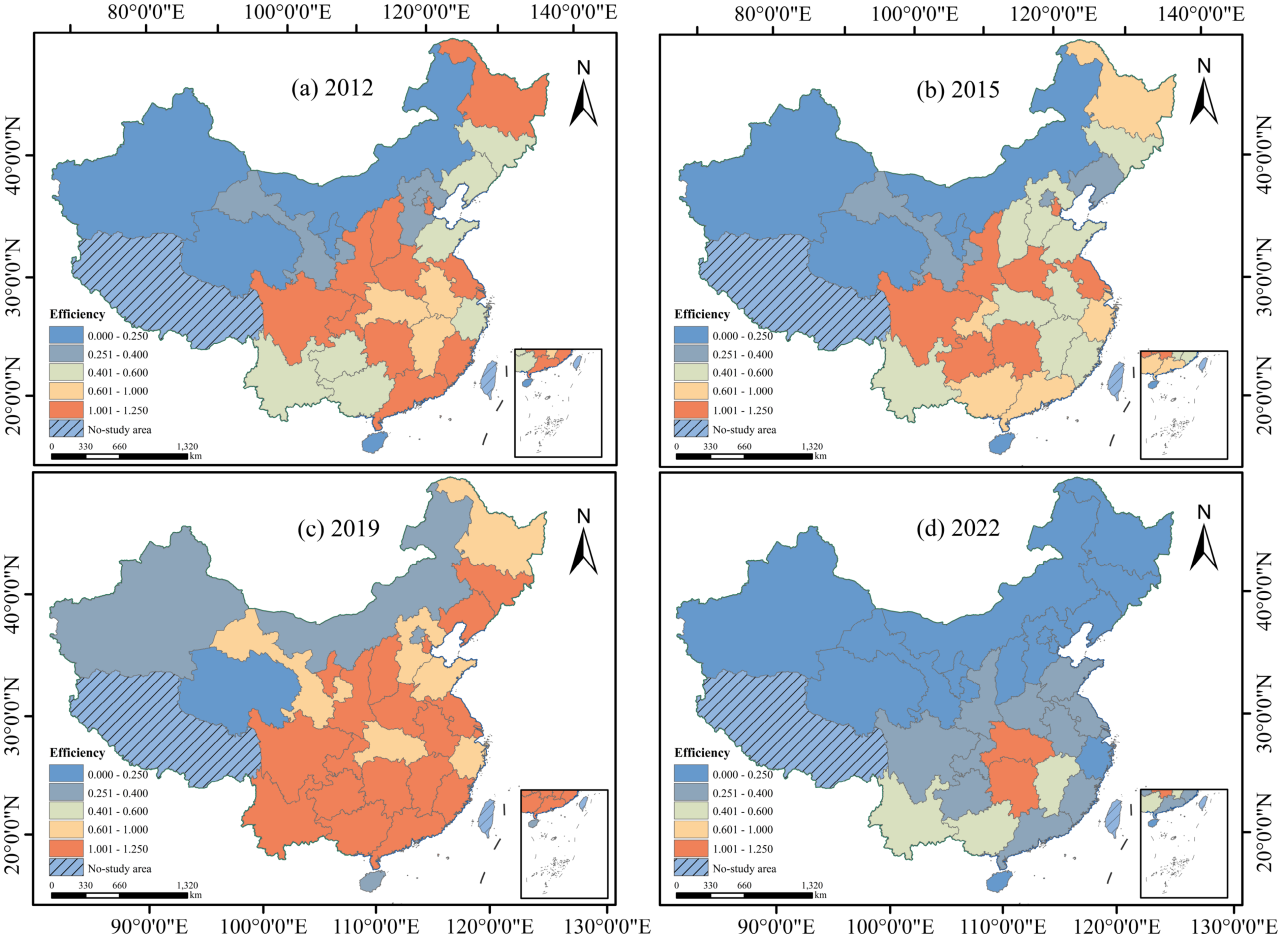
Spatial distribution of CHECTE by province.

Based on the timeline analysis of China’s Five-Year Plans, key policies, and external shocks discussed earlier, two distinct turning points can be identified: 2015 and 2019 mark pivotal moments in the shift of efficiency. And using the first and last years as reference periods, ArcGIS was employed to map the provincial distribution of CHECTE values for 2012, 2015, 2019, and 2022. The results are illustrated in Figure 6. It was found that during the decline period, the distribution of efficient regions contracted toward the central and western inland areas, while during the rise period, the distribution of efficient regions re-expanded toward the southwest and southeast, and also matches the characteristics of the average efficiency distribution.

Additionally, the distribution maps show that high-efficiency provinces exhibit spatial clustering (Figures 5, 6). For example, the Chengdu-Chongqing Twin Cities Economic Circle is starting to emerge as a potential high-efficiency region, while high-efficiency clusters are forming in areas such as Fujian and Guangdong, the Yangtze River Delta, and the Yellow River Golden Triangle Region of Shanxi-Shaanxi-Henan. This emphasizes the positive effects of regional policy synergies.

### 4.2 Tobit regression analysis

#### 4.2.1 Regression results

The multicollinearity test shows that all variables have VIF values below 5, indicating no serious multicollinearity. Based on the Hausman test, a fixed-effects panel Tobit regression was performed, with the results presented in Table 3 (Model 1).

**Table 3:** Tobit regression results.

Variable	Model (1) Tobit regression	Model (2) Excluding data of 2020	Model (3) OLS regression
TC	0.117*** (3.830)	0.114*** (3.700)	0.124*** (3.410)
IN	0.100*** (4.900)	0.092*** (4.220)	0.099*** (3.870)
PE	−0.388* (−1.940)	−0.475** (−2.210)	−0.514** (−2.040)
CD	0.072 (1.150)	0.056 (0.820)	0.060 (0.760)
HE	0.106** (2.050)	0.153*** (2.840)	0.166*** (2.620)
TI	0.371*** (3.720)	0.421*** (4.130)	0.455*** (3.810)
Constant	−0.762** (−1.99)	−1** (−2.55)	−1** (−2.35)
Individual effect	Control	Control	Control
Time effect	Control	Control	Control

*, **, and *** indicate significance at the 10, 5, and 1% levels, respectively.

The regression coefficient of TC is 0.117 and is statistically significant at the 1% level, indicating a strong positive effect. As a fundamental support for tourism, transportation accessibility enables tourists to reach their destinations more easily. In particular, improved transport infrastructure helps unlock the potential of lesser-known areas rich in cultural heritage, making such resources more visible and usable (9). Enhanced transportation and passenger mobility connect remote cultural sites to broader networks, attracting more tourists, stimulating local consumption. This, in turn, deepens the integration of culture, tourism, and regional economic development. Notably, infrastructure-related policies have further reinforced these effects—for instance, Guizhou’s completion of the “high-speed to counties” project in 2015 significantly increased both passenger capacity and tourism reception capabilities, with CHECTE values remaining above 1 thereafter.

The regression coefficient of IN is 0.10, significant at the 1% level, indicating a strong positive effect. This highlights that a higher degree of informatization—represented by the length of fiber-optic cable—facilitates information dissemination and improves service convenience. In response, many regions have actively developed smart tourism systems and digital platforms that allow tourists to book online easily. These advancements significantly enhance the overall visitor experience and improve operational efficiency across the cultural tourism industry. For example, the Sichuan Information and Communication Industry Development Report (2023) notes that the length of fiber-optic cables in Sichuan ranks first in central and western China and second nationwide. This robust digital infrastructure supports platforms such as “One Mobile Phone Touring Sichuan.” Meanwhile, the CHECTE of Sichuan has been in a state of super efficiency all year round during the research period.

The regression coefficient of PE is −0.388, significant at the 10% level, indicating a negative impact. This suggests diminishing marginal returns on government financial expenditures in the cultural tourism sector. Although public spending on culture, tourism, and related services—especially in infrastructure, promotion, and service capacity—should theoretically enhance industry development (5), practical inefficiencies often limit its effectiveness. In some regions, increased fiscal input has not been matched by targeted investments in key areas, such as publicity or infrastructure, leading to suboptimal outcomes. As Sulaiman (53) noted, tourism themes must align with tourists’ cultural interests to be effective; otherwise, promotion may fail to generate appeal. Similarly, Huang et al. (54) argued that ineffective promotional activities can result in financial waste. Project performance reports^1^ further show imbalances between resource development and actual operations, reinforcing the conclusion that financial inputs in cultural tourism must be more efficient and precisely targeted.

The regression coefficient of CD is 0.072, but it does not pass the significance test. While this indicator reflects residents’ willingness and capacity to spend on cultural and recreational activities, significant regional differences in consumption patterns lead to heterogeneity in its impact. In some regions, high per capita recreational spending does not translate into upgrades in cultural tourism linked to cultural heritage. For instance, although Beijing reports high recreational consumption, high input costs and redundancy may reduce efficiency. In contrast, provinces like Yunnan and Guangxi, despite lower spending levels, leverage natural and cultural resources to operate more efficiently and realize effective transformation in cultural tourism.

In contrast, the regression coefficient of HE is 0.106 and is significant at the 5% level, indicating a clear positive impact. A higher proportion of educated individuals provides stronger intellectual and talent support for cultural tourism development, consistent with findings by Yolal and Negrusa (50), Huang et al. (54), and Del Barrio-Tellado et al. (12). Through participation in cultural tourism planning, heritage interpretation, and cultural product design, universities and their graduates contribute to the development of innovative models such as “university + cultural tourism,” thereby enhancing both quality and efficiency in the industry.

The regression coefficient of TI is 0.371, significant at the 1% level, and shows the strongest positive impact among all variables, highlighting the importance in enhancing the CHECTE. Key contributions include the development of digital culture tourism service platforms, the improvement of digital services at museums and scenic spots, and the application of technology in the preservation and transmission of cultural heritage. Emerging technologies such as VR and AR support immersive cultural experiences, while big data analytics enable precise identification of tourist preferences and targeted marketing strategies. These advancements collectively promote the digital transformation of cultural heritage embedded cultural tourism, becoming a key driver of efficiency and high-quality development in the sector.

#### 4.2.2 Robustness test

To enhance the robustness of results, the following methods were selected for testing: (1) Re-running the Tobit regression after removing the 2020 data, which was significantly affected by the COVID-19 pandemic; (2) Applying OLS regression, with the regression results displayed in Table 3.

The findings indicate that after adjusting the time window period and regression method, the significance of the variables is maintained, and the direction of the influence remains unchanged. In terms of effect magnitude, most influencing factors remain consistent with the original model. Notably, the absolute value of the PE coefficient increases in Model 2 after excluding 2020 data, suggesting that the COVID-19 pandemic had a complex impact on the cultural tourism industry. This may have disrupted the normal functioning of policy mechanisms and masked the inefficiencies of policy expenditures. In Model 3, based on ordinary panel regression, the coefficients for HE and TI are larger, indicating that their marginal effects are more pronounced in a linear framework.

### 4.3 Analysis of regional heterogeneity

To better understand the regional differences in influencing factors, this study conducts separate regression analyses for provinces in different regions, with the results presented in Table 4.

**Table 4:** Regional heterogeneity regression results.

Variable	The Eastern	The Central	The Western
TC	0.072** (2.030)	−0.369*** (−2.780)	0.432*** (6.640)
IN	0.049 (1.180)	0.153** (2.580)	0.027 (0.840)
PE	−0.571** (−2.170)	2.392** (2.110)	−0.194 (−0.570)
CD	0.128* (1.830)	−0.182 (−0.590)	0.371 (1.410)
HE	−0.080 (−1.010)	−0.053 (−0.410)	0.221*** (2.700)
TI	0.334** (2.470)	0.320 (0.850)	−0.550* (−1.750)
Constant	0.471 (0.87)	0.631* (1.90)	−0.638** (−2.12)
Individual effect	Control	Control	Control
Time effect	Control	Control	Control

*, **, and *** indicate significance at the 10, 5, and 1% levels, respectively.

In the eastern region, TC, PE, CD, and TI are statistically significant. The eastern region has a developed economy and a concentration of high-tech industries (55, 56), resulting in the strongest positive impact of TI on promoting CHECTE, with a regression coefficient of 0.334. Conversely, in the western region, TI shows a negative effect with a regression coefficient of −0.550, confirming findings by scholars such as Chen et al. (45), who argue that TI can only stimulate development once a region has reached a certain economic threshold. The positive effect of CD is significant only in the east, with a regression coefficient of 0.128, as economically advanced regions tend to generate greater demand for leisure activities, thus more directly influencing CHECTE. The PE exhibits a negative impact in eastern regions, with a regression coefficient of −0.571. This indicates diminishing marginal returns on public spending in cultural tourism, suggesting potential inefficiencies in fiscal allocation. Taking Liaoning Province as an example, in 2021—a pivotal year of recovery following the COVID-19 pandemic—expenditures on culture, tourism, sports, and media increased by 12% year-on-year, exceeding the national average growth rate of 9%. Notably, cultural tourism promotion projects accounted for 64.75% of total expenditures, reflecting strong policy support. However, the CHECTE remained low at 0.2309, failing to generate positive feedback.

In the central region, TC, IN, and PE all passed the significance test. This suggests that digital infrastructure and policy support significantly promote CHECTE, with PE having the strongest positive effect. The central region’s development has benefited from the “Rise of Central China” strategy (55, 57), where policy-driven industrial restructuring is the key driving force for advancing cultural tourism (45). Effective policy guidance is crucial for the central region, as evidenced by the significant positive impacts of the PE and IN, with regression coefficients of 0.153 and 2.392, respectively, where PE is the most substantial. However, TC shows a negative effect, with a regression coefficient of −0.369. As a key national transit corridor, the central region experiences high passenger flow but short tourist stays. The expansion of high-speed rail has made same-day travel more convenient, reducing overnight stays (58). Studies also show that high-speed rail may create a “tunnel effect,” favoring core cities while marginalizing surrounding areas (59, 60). This effect is more pronounced in the central region due to its transportation hub status. Furthermore, transportation planning here often prioritizes logistics and freight. For instance, Hubei’s plan emphasizes rail freight and multimodal transport, yet dedicates limited attention to passenger services. This mismatch may weaken the synergy between transport infrastructure and CHECTE.

For the western region, TC, HE, and TI pass the significance test. In western regions with relatively balanced endowments, the spillover effects of high-speed rail are stronger, helping to narrow interregional disparities (61). Correspondingly, TC has a most significant positive impact with a regression coefficient of 0.432, as most western regions are characterized by high terrain, complex climates, and slow economic development, making their cultural tourism development more reliant on resource endowment (62), and cultural heritage is one of the significant advantages of this type of region, as long as the infrastructure is done well to eliminate the transportation barriers, give play to the advantages and attract more tourists. On this basis, in order to activate the cultural tourism resources, it is necessary to actively learn from advanced experiences to optimize the cultural tourism development mode and investment efficiency (45). The demand for high-quality talents and professionals arises, so the HE has an obvious positive effect on CHECTE in the west, with a regression coefficient of 0.221.

## 5 Conclusion

### 5.1 Key findings

This study employs the global reference Super-SBM-DEA model to evaluate the CHECTE across 30 Chinese provinces from 2012 to 2022. The results identify a three-stage temporal trend of “declining-rising-declining” over the study period. After dropping to 0.5829 in 2015, CHECTE peaked at 0.8511 in 2019, before declining sharply under the impact of the COVID-19 pandemic. Spatially, the efficiency distribution exhibits a distinct deviation from conventional regional economic gradients. Several eastern provinces did not display pronounced advantages, whereas certain central and western provinces achieved comparatively high levels of efficiency. The central region benefited most from cultural heritage embedding, achieving an average efficiency of 0.7499—higher than the overall average of 0.5843 and the eastern average of 0.5746. Tobit regression further elucidates distinct regional drivers. However, the effects of these factors differ significantly across eastern, central, and western regions. The eastern region is primarily influenced by TI and CD, with regression coefficients of 0.334 and 0.128, respectively. The central region responds strongly to PE and IN, with regression coefficients of 2.392 and 0.153, respectively. In the west, the dominant factors are TC and HE, with regression coefficients of 0.432 and 0.221, respectively. These findings underscore the need for region-specific policies tailored to these differential influences.

### 5.2 Policy implications

China’s vast territory exhibits substantial disparities in natural environments, resource endowments, and economic development. It is therefore crucial to develop targeted strategies based on the spatio-temporal evolution characteristics, driving mechanisms, and regional heterogeneity of CHECTE during the study period.

Firstly, analysis of regional influencing factors reveals that TI and CD have played a particularly positive role in the eastern region. This suggests the need to further promote innovation in consumption scenarios and accelerate digital transformation. A notable example is Jiangsu Province, which recorded the highest average CHECTE value of 0.8616 in the eastern region, with scores consistently above 1 between 2012 and 2019. This sustained high efficiency stems from its early commitment—starting in 2012—to integrating culture and technology. Through a series of policy measures, Jiangsu actively developed the smart tourism platform “Su Xin You,” introduced VR technologies in museums, and stimulated cultural tourism consumption through technological advances. By strengthening such innovations and advancing digital transformation, the eastern region can more effectively unlock the socioeconomic potential of cultural heritage tourism and achieve high-quality development.

In the central region, PE plays a significant role, with successful projects like the “South Anhui International Cultural Tourism Demonstration Zone,” contributing to Anhui’s efficiency rise from 0.5789 in 2015 to 1.1196 in 2016. The plan was formally implemented in 2014, actively allocating funds and improving infrastructure. In 2016, a special initiative was launched to promote “Anhui Culture” tourism, focusing on deepening the integration and upgrading of cultural tourism industries. This provided replicable pathways for relevant regions, prompting central China to proactively deploy the development and operation of advantageous projects.

For the western region, addressing transportation and infrastructure deficiencies is a priority. Several key policies have directly impacted transportation in the region. For instance, Guizhou’s completion of its “Expressway Access for Every County” policy in 2015 significantly enhanced passenger transport capacity and tourism reception capabilities. The CHECTE rose from 0.4406 in 2014 to 1.0139 in 2015, underscoring the importance of targeted policies. Improved transportation networks can reduce the flow costs of resources and people. Additionally, cultivating high-quality talent and learning from advanced experiences will optimize CHECTE, unlocking the potential of unique cultural and ecological tourism resources.

Additionally, it is important to note that the efficiency decline caused by the pandemic’s impact underscores the need for cultural tourism to enhance its resilience to risks. This can be achieved through innovations like cloud exhibitions, short-distance micro-vacations, and leveraging digital technologies to offer new cultural tourism experiences, thus maintaining resilience against external shocks. Meanwhile, the spatial distribution of high-efficiency provinces exhibits a certain level of spatial autocorrelation, with high-efficiency clusters formed in regions such as Fujian and Guangdong, the Chengdu-Chongqing Twin Cities, and the Yellow River Golden Triangle Region of Shanxi-Shaanxi-Henan. To foster continued cultural and tourism linkage across economic zones, it is essential to leverage spatial correlations and promote synergistic effects.

### 5.3 Limitations and prospects

The rapid development of tourism may have certain negative impacts, such as ecological and carbon emission dangers, as well as the destruction of cultural integrity and originality. In fact, having unique cultural heritage often becomes the main motivation for tourism activities, but the development and construction of infrastructure may threaten the integrity of heritage, so the subsequent assessment system can consider non-desired output factors.

Although panel Tobit regression is employed here to explore influencing factors and their regional heterogeneity, potential synergies between cultural resources and tourism development may exist, particularly as more emphasis is placed on the organic integration of traditional cultures such as ICH into modern life. Future research could further investigate the coupling mechanisms between cultural heritage resource systems and tourism development systems to deepen understanding of their interactive dynamics and coordinated growth.

In the context of common prosperity, the development of cultural tourism stimulates the consumer market, which in turn promotes the local economy. However, in terms of resource perspective, irrational development and utilization may damage the ecological and cultural authenticity, affecting the effect of protection and inheritance. Thus, the linkage between the advancement of cultural tourism and the safeguarding and continuation of cultural heritage merits deeper study, with emphasis on specific heritage forms and micro-level case analyses.

## Data Availability

The raw data supporting the conclusions of this article will be made available by the authors, without undue reservation.

## Footnotes

1
whly.ln.gov.cn
